# Supplementary figures and images for: Stochastic model of Alzheimer’s disease progression using two-state Markov chains
Source: PLoS One. 2024 Jan 19;19(1):e0295578. doi: 10.1371/journal.pone.0295578 (PMC10798450; doi:10.1371/journal.pone.0295578)

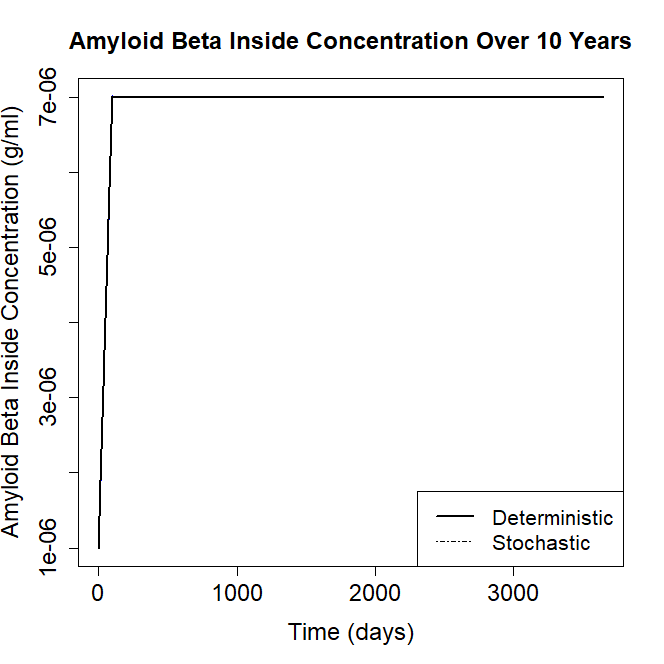

Supplement: S1 Fig — (PNG) [file pone.0295578.s001.png]

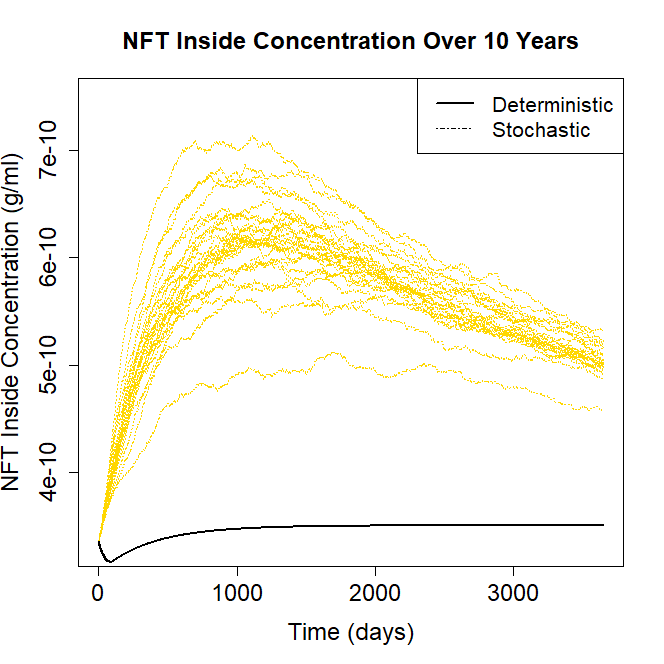

Supplement: S2 Fig — (PNG) [file pone.0295578.s002.png]

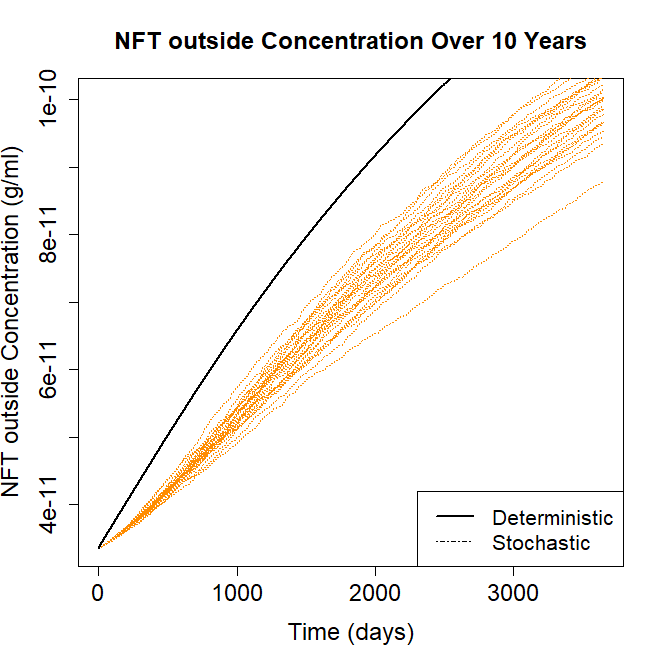

Supplement: S3 Fig — (PNG) [file pone.0295578.s003.png]

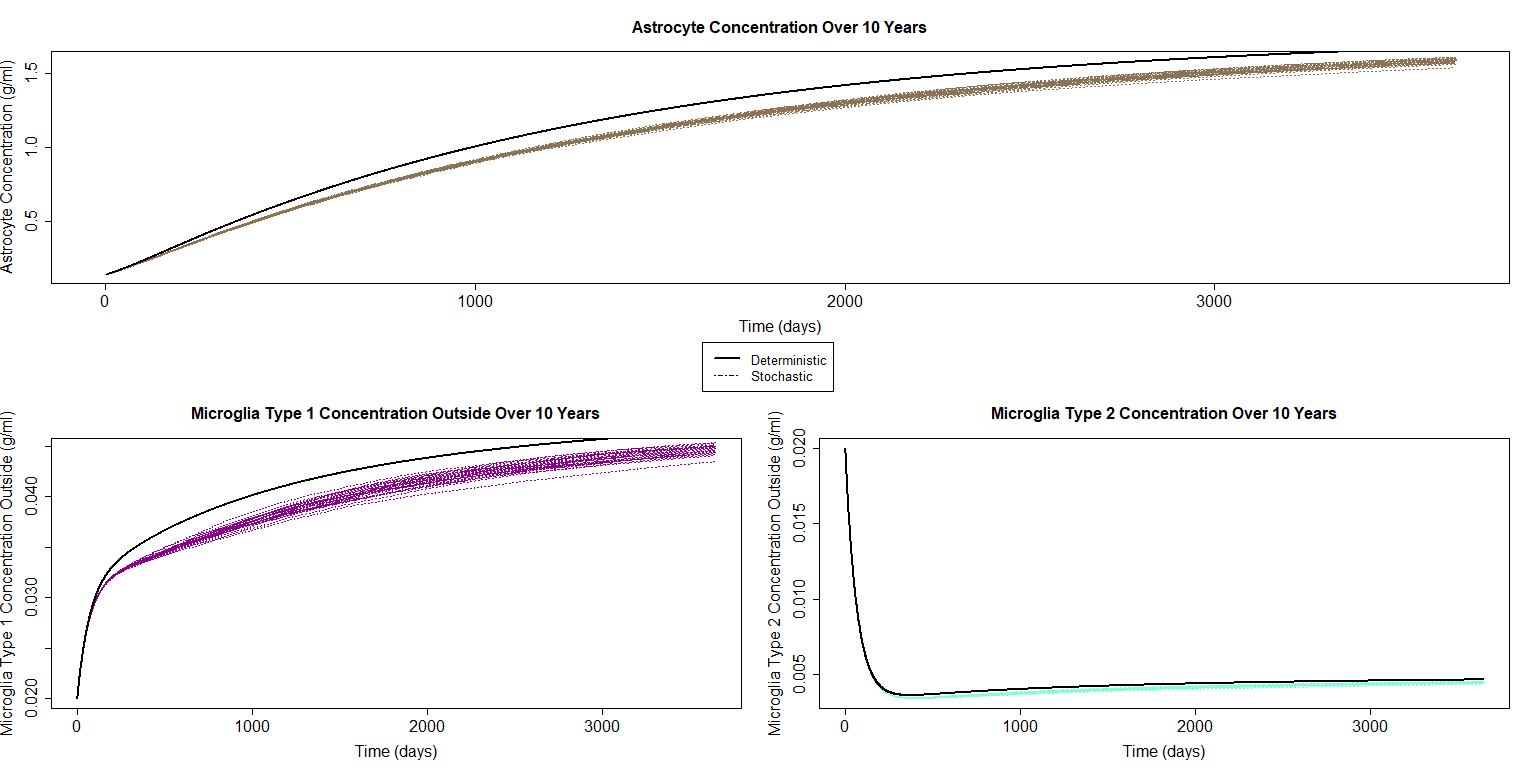

Supplement: S4 Fig — (PNG) [file pone.0295578.s004.png]

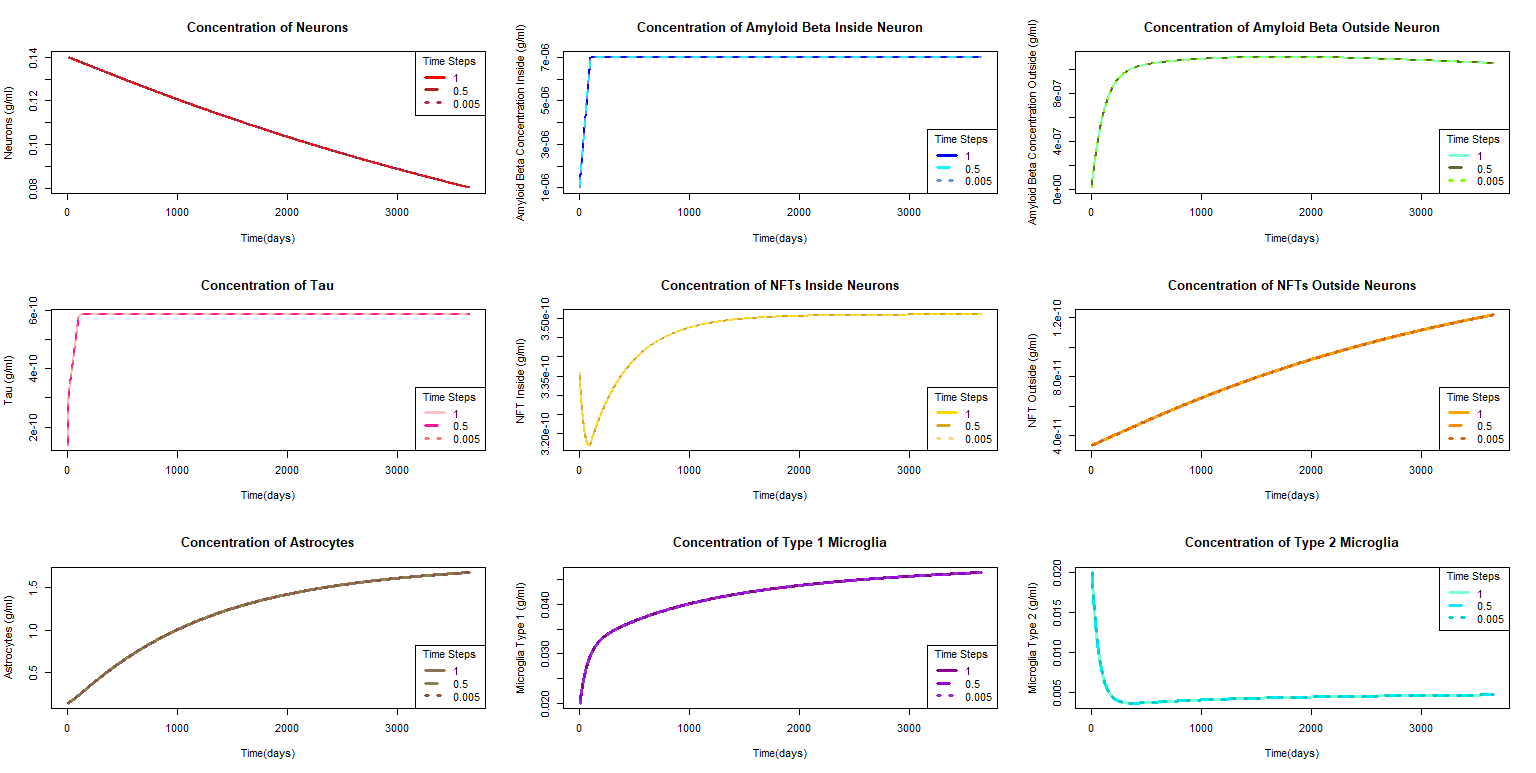

Supplement: S5 Fig — (PNG) [file pone.0295578.s005.png]
