# Supplementary material for: Stochastic model of Alzheimer’s disease progression using two-state Markov chains
Source: PLoS One. 2024 Jan 19;19(1):e0295578. doi: 10.1371/journal.pone.0295578 (PMC10798450; doi:10.1371/journal.pone.0295578)
Supplement: Attachment — Submitted filename: Reviwer comments.docx [file pone.0295578.s006.docx]

Dear Editor

The manuscript entitled “Effect of MEF2A and SLC22A3-LPAL2-LPA Gene Polymorphisms on Warfarin Sensitivity and Responsiveness in Jordanian Cardiovascular Patient” is an interesting piece of work which investigates the response of Jordanian Cardiovascular Patients with different genetic polymorphisms to the treatment of anticoagulant drug Warfarin. The effect of most drugs often varies among the patients suffering with the same disease. One of the highly belief reason for this is the differences in genetic composition among different individuals. Therefore this piece of information will be beneficial for the Cardiovascular Patients and further research in this field. However I have few comments. I would like to accept the article for publication in PLOS ONE after the revision of the manuscript by the authors.

Comments

1. In the Result section line 23, SLC22A3-LPAL2-LPA rs10755578 was mentioned to be associated whereas in the conclusion section, line 31, different SNP rs10455872 was mentioned to be associated. Authors may clarify this.
2. In the method section, line 93-95, age range is not clearly mentioned. I would request authors to clearly mention the age range of the cases and controls within these lines.
3. In Table S7, I would suggest authors to add number of cases and controls for each haplotype as well.
4. Authors need to mention how the responsiveness of the patients or sensitivity of Warfarin has been measured.
5. Authors also should mention about drug doses received by the patients because it can relate to the sensitivity of the drug.
