# Supplementary material for: Stochastic model of Alzheimer’s disease progression using two-state Markov chains
Source: PLoS One. 2024 Jan 19;19(1):e0295578. doi: 10.1371/journal.pone.0295578 (PMC10798450; doi:10.1371/journal.pone.0295578)
Supplement: Attachment — Submitted filename: Parks 2023 PLoS One_ Response to Reviewers.docx [file pone.0295578.s007.docx]

**Reviewer Comments in Red**

Reviewer #1: The paper Stochastic Model of Alzheimer’s Disease Progression Using Two-State Markov Chains is well organized and build on good data base. The methodology is sound, and the analysis is well done. The analysis of data in result section is clear. The discussion and conclusions are appropriate. The paper is suitable for publication in the present form.

Thank you very much for your kind words, much effort was put into clearly presenting the model.

Reviewer #2: The manuscript entitled “Stochastic model of Alzheimer’s disease progression using two-state Markov chain” is an interesting article that will benefit in the field of Alzheimer’s research. Moreover, the interaction between microglia and astrocytes in neurodegeneration and cognitive impairment is one of the immerging researches in the dementia research. I am expert in statistics and computational modeling. The manuscript is nicely written.

Thank you, I am happy you see the value in the research presented in my manuscript.

Nonetheless, I have this one major issue as a biologist, in regard to its application, the use of this model using various data generated from biological researches. I hope it will not be difficult for the authors to explain this in this manuscript.

All chemical half reactions and associated parameters were derived from existing biological studies in the original Hao and Friedman manuscript which also described the parameterization of the model in the Appendix section of the manuscript. The research used to develop these parameters dates back to 1975 and is spread between around 28 different publications (<https://doi.org/10.1186/s12918-016-0348-2>, [The density of synapses and neurons in normal, mentally defective ageing human brains. - Abstract - Europe PMC](https://europepmc.org/article/med/123474)).

In the re-submission, we now address this in the revised Variables & Parameters section of Methods We say:

The nine variables on which the model relies are shown in Table 1, the table also displays the initial values used for both models, these values are those used by Hao and Friedman (2016). The variables used in the model were chosen based on the prevailing theory that the driving force behind Alzheimer’s Disease is the formation of Amyloid plaques and Neurofibrillary tangles. The equations and parameters used in both the deterministic and stochastic models were taken directly from Hao and Friedmans work or a modified version of these values (2016). The parameterization of Hao and Friedman’s deterministic model using biological data was done in exemplary detail in the Appendix of the original manuscript and references 28 publications dating back to 1975 (2016). A litany of methods were utilized, including biophysical and biochemical measurements, histopathology on normal and diseased human cadavers, ImmunoHistoChemistry (IHC), NMR spectroscopy, transgenic mouse constructs, and pulse-chase labeling, to find this data (Hao & Friedman, 2016). Despite the variety of methods and effort committed to estimating these parameters there is still error when estimating these parameters. This inaccuracy does limit the applicability of models such as these.

In Table. 2 values marked with asterisks are the same as those used by Hao and Friedman. Those without asterisks were modified by us. Only four parameters were modified in the newly proposed model as described here. The parameter Nd represents the rate of normal neuronal loss with age; after the age of forty, neurons begin to die at ~5% each year. The parameter MA is a combination of two separate parameters used by Hao & Friedman: the production of TNF-alpha inflammatory microglia and the production of astrocytes by TNF72 alpha. Because we omitted TNF-alpha in the model, we multiplied the two values to bridge the gap between the astrocytes and inflammatory microglia, both of which are elements of our model. M1 and M2 represent the effect of inflammation on inflammatory microglia M1 and its effect on anti-inflammatory microglia M2.
