# Supplementary material for: Stochastic model of Alzheimer’s disease progression using two-state Markov chains
Source: PLoS One. 2024 Jan 19;19(1):e0295578. doi: 10.1371/journal.pone.0295578 (PMC10798450; doi:10.1371/journal.pone.0295578)
Supplement: Peer review history

# Peer review: Decision Letter 0

**DOI:** 10.1371/journal.pone.0295578.r001

**Authors:** Asem Surindro Singh

30 Oct 2023

PONE-D-23-15181Stochastic Model of Alzheimer’s Disease Progression Using Two-State Markov ChainsPLOS ONE

Dear Dr. Meaghan Elizabeth Parks,

Thank you for submitting your manuscript to PLOS ONE. After careful consideration, we feel that it has merit but does not fully meet PLOS ONE’s publication criteria as it currently stands. Therefore, we invite you to submit a revised version of the manuscript that addresses the points raised during the review process.

Please submit your revised manuscript by Dec 14 2023 11:59PM. If you will need more time than this to complete your revisions, please reply to this message or contact the journal office at plosone@plos.org. When you're ready to submit your revision, log on to https://www.editorialmanager.com/pone/ and select the 'Submissions Needing Revision' folder to locate your manuscript file.

Please include the following items when submitting your revised manuscript:

- A rebuttal letter that responds to each point raised by the academic editor and reviewer(s). You should upload this letter as a separate file labeled 'Response to Reviewers'.
- A marked-up copy of your manuscript that highlights changes made to the original version. You should upload this as a separate file labeled 'Revised Manuscript with Track Changes'.
- An unmarked version of your revised paper without tracked changes. You should upload this as a separate file labeled 'Manuscript'.

If you would like to make changes to your financial disclosure, please include your updated statement in your cover letter. Guidelines for resubmitting your figure files are available below the reviewer comments at the end of this letter.

If applicable, we recommend that you deposit your laboratory protocols in protocols.io to enhance the reproducibility of your results. Protocols.io assigns your protocol its own identifier (DOI) so that it can be cited independently in the future. For instructions see: https://journals.plos.org/plosone/s/submission-guidelines#loc-laboratory-protocols. Additionally, PLOS ONE offers an option for publishing peer-reviewed Lab Protocol articles, which describe protocols hosted on protocols.io. Read more information on sharing protocols at https://plos.org/protocols?utm_medium=editorial-email&utm_source=authorletters&utm_campaign=protocols.

We look forward to receiving your revised manuscript.

Kind regards,

Asem Surindro Singh, Ph.D

Academic Editor

PLOS ONE

Journal Requirements:

When submitting your revision, we need you to address these additional requirements.

1. Please ensure that your manuscript meets PLOS ONE's style requirements, including those for file naming. The PLOS ONE style templates can be found at

https://journals.plos.org/plosone/s/file?id=wjVg/PLOSOne_formatting_sample_main_body.pdf and

https://journals.plos.org/plosone/s/file?id=ba62/PLOSOne_formatting_sample_title_authors_affiliations.pdf.

2. In your Data Availability statement, you have not specified where the minimal data set underlying the results described in your manuscript can be found. PLOS defines a study's minimal data set as the underlying data used to reach the conclusions drawn in the manuscript and any additional data required to replicate the reported study findings in their entirety. All PLOS journals require that the minimal data set be made fully available. For more information about our data policy, please see http://journals.plos.org/plosone/s/data-availability.

"Upon re-submitting your revised manuscript, please upload your study’s minimal underlying data set as either Supporting Information files or to a stable, public repository and include the relevant URLs, DOIs, or accession numbers within your revised cover letter. For a list of acceptable repositories, please see http://journals.plos.org/plosone/s/data-availability#loc-recommended-repositories. Any potentially identifying patient information must be fully anonymized.

Important: If there are ethical or legal restrictions to sharing your data publicly, please explain these restrictions in detail. Please see our guidelines for more information on what we consider unacceptable restrictions to publicly sharing data: http://journals.plos.org/plosone/s/data-availability#loc-unacceptable-data-access-restrictions. Note that it is not acceptable for the authors to be the sole named individuals responsible for ensuring data access.

We will update your Data Availability statement to reflect the information you provide in your cover letter.

3. Please review your reference list to ensure that it is complete and correct. If you have cited papers that have been retracted, please include the rationale for doing so in the manuscript text, or remove these references and replace them with relevant current references. Any changes to the reference list should be mentioned in the rebuttal letter that accompanies your revised manuscript. If you need to cite a retracted article, indicate the article’s retracted status in the References list and also include a citation and full reference for the retraction notice.

[Note: HTML markup is below. Please do not edit.]

Reviewers' comments:

Reviewer's Responses to Questions

**Comments to the Author**

1. Is the manuscript technically sound, and do the data support the conclusions?

The manuscript must describe a technically sound piece of scientific research with data that supports the conclusions. Experiments must have been conducted rigorously, with appropriate controls, replication, and sample sizes. The conclusions must be drawn appropriately based on the data presented. 

Reviewer #1: Yes

Reviewer #2: Yes

**********

2. Has the statistical analysis been performed appropriately and rigorously? 

Reviewer #1: Yes

Reviewer #2: Yes

**********

3. Have the authors made all data underlying the findings in their manuscript fully available?

The PLOS Data policy requires authors to make all data underlying the findings described in their manuscript fully available without restriction, with rare exception (please refer to the Data Availability Statement in the manuscript PDF file). The data should be provided as part of the manuscript or its supporting information, or deposited to a public repository. For example, in addition to summary statistics, the data points behind means, medians and variance measures should be available. If there are restrictions on publicly sharing data—e.g. participant privacy or use of data from a third party—those must be specified.

Reviewer #1: Yes

Reviewer #2: Yes

**********

4. Is the manuscript presented in an intelligible fashion and written in standard English?

PLOS ONE does not copyedit accepted manuscripts, so the language in submitted articles must be clear, correct, and unambiguous. Any typographical or grammatical errors should be corrected at revision, so please note any specific errors here.

Reviewer #1: Yes

Reviewer #2: Yes

**********

5. Review Comments to the Author

Please use the space provided to explain your answers to the questions above. You may also include additional comments for the author, including concerns about dual publication, research ethics, or publication ethics. (Please upload your review as an attachment if it exceeds 20,000 characters)

Reviewer #1: The paper Stochastic Model of Alzheimer’s Disease Progression Using Two-State Markov Chains is well organized and build on good data base. The methodology is sound, and the analysis is well done. The analysis of data in result section is clear. The discussion and conclusions are appropriate. The paper is suitable for publication in the present form.

Reviewer #2: The manuscript entitled “Stochastic model of Alzheimer’s disease progression using two-state Markov chain” is an interesting article that will benefit in the field of Alzheimer’s research. Moreover, the interaction between microglia and astrocytes in neurodegeneration and cognitive impairment is one of the immerging researches in the dementia research. I am expert in statistics and computational modeling. The manuscript is nicely written.

Nonetheless, I have this one major issue as a biologist, in regard to its application, the use of this model using various data generated from biological researches. I hope it will not be difficult for the authors to explain this in this manuscript.

**********

6. PLOS authors have the option to publish the peer review history of their article (what does this mean?). If published, this will include your full peer review and any attached files.

If you choose “no”, your identity will remain anonymous but your review may still be made public.

**Do you want your identity to be public for this peer review?** For information about this choice, including consent withdrawal, please see our Privacy Policy.

Reviewer #1: **Yes: **Jagannatha Rao KS

Reviewer #2: **Yes: **Dr. Machathoibi Takhellambam Chanu

**********

[NOTE: If reviewer comments were submitted as an attachment file, they will be attached to this email and accessible via the submission site. Please log into your account, locate the manuscript record, and check for the action link "View Attachments". If this link does not appear, there are no attachment files.]

While revising your submission, please upload your figure files to the Preflight Analysis and Conversion Engine (PACE) digital diagnostic tool, https://pacev2.apexcovantage.com/. PACE helps ensure that figures meet PLOS requirements. To use PACE, you must first register as a user. Registration is free. Then, login and navigate to the UPLOAD tab, where you will find detailed instructions on how to use the tool. If you encounter any issues or have any questions when using PACE, please email PLOS at figures@plos.org. Please note that Supporting Information files do not need this step.

Attachment Submitted filename: Reviwer comments.docx Click here for additional data file.

# Author comment: Author response to Decision Letter 0

**DOI:** 10.1371/journal.pone.0295578.r002

17 Nov 2023

Reviewer Comments in Red

Reviewer #1: The paper Stochastic Model of Alzheimer’s Disease Progression Using Two-State Markov Chains is well organized and build on good data base. The methodology is sound, and the analysis is well done. The analysis of data in result section is clear. The discussion and conclusions are appropriate. The paper is suitable for publication in the present form.

Thank you very much for your kind words, much effort was put into clearly presenting the model.

Reviewer #2: The manuscript entitled “Stochastic model of Alzheimer’s disease progression using two-state Markov chain” is an interesting article that will benefit in the field of Alzheimer’s research. Moreover, the interaction between microglia and astrocytes in neurodegeneration and cognitive impairment is one of the immerging researches in the dementia research. I am expert in statistics and computational modeling. The manuscript is nicely written.

Thank you, I am happy you see the value in the research presented in my manuscript.

Nonetheless, I have this one major issue as a biologist, in regard to its application, the use of this model using various data generated from biological researches. I hope it will not be difficult for the authors to explain this in this manuscript.

All chemical half reactions and associated parameters were derived from existing biological studies in the original Hao and Friedman manuscript which also described the parameterization of the model in the Appendix section of the manuscript. The research used to develop these parameters dates back to 1975 and is spread between around 28 different publications (https://doi.org/10.1186/s12918-016-0348-2, The density of synapses and neurons in normal, mentally defective ageing human brains. - Abstract - Europe PMC).

In the re-submission, we now address this in the revised Variables & Parameters section of Methods We say:

The nine variables on which the model relies are shown in Table 1, the table also displays the initial values used for both models, these values are those used by Hao and Friedman (2016). The variables used in the model were chosen based on the prevailing theory that the driving force behind Alzheimer’s Disease is the formation of Amyloid plaques and Neurofibrillary tangles. The equations and parameters used in both the deterministic and stochastic models were taken directly from Hao and Friedmans work or a modified version of these values (2016). The parameterization of Hao and Friedman’s deterministic model using biological data was done in exemplary detail in the Appendix of the original manuscript and references 28 publications dating back to 1975 (2016). A litany of methods were utilized, including biophysical and biochemical measurements, histopathology on normal and diseased human cadavers, ImmunoHistoChemistry (IHC), NMR spectroscopy, transgenic mouse constructs, and pulse-chase labeling, to find this data (Hao & Friedman, 2016). Despite the variety of methods and effort committed to estimating these parameters there is still error when estimating these parameters. This inaccuracy does limit the applicability of models such as these.

In Table. 2 values marked with asterisks are the same as those used by Hao and Friedman. Those without asterisks were modified by us. Only four parameters were modified in the newly proposed model as described here. The parameter Nd represents the rate of normal neuronal loss with age; after the age of forty, neurons begin to die at ~5% each year. The parameter MA is a combination of two separate parameters used by Hao & Friedman: the production of TNF-alpha inflammatory microglia and the production of astrocytes by TNF72 alpha. Because we omitted TNF-alpha in the model, we multiplied the two values to bridge the gap between the astrocytes and inflammatory microglia, both of which are elements of our model. M1 and M2 represent the effect of inflammation on inflammatory microglia M1 and its effect on anti-inflammatory microglia M2.

Attachment Submitted filename: Parks 2023 PLoS One_ Response to Reviewers.docx Click here for additional data file.

# Editor report: Decision Letter 1

**DOI:** 10.1371/journal.pone.0295578.r003

**Authors:** Asem Surindro Singh

27 Nov 2023

Stochastic Model of Alzheimer’s Disease Progression Using Two-State Markov Chains

PONE-D-23-15181R1

Dear Dr. Meaghan Elizabeth Parks,

We’re pleased to inform you that your manuscript has been judged scientifically suitable for publication and will be formally accepted for publication once it meets all outstanding technical requirements.

Within one week, you’ll receive an e-mail detailing the required amendments. When these have been addressed, you’ll receive a formal acceptance letter and your manuscript will be scheduled for publication.

An invoice for payment will follow shortly after the formal acceptance. To ensure an efficient process, please log into Editorial Manager at http://www.editorialmanager.com/pone/, click the 'Update My Information' link at the top of the page, and double check that your user information is up-to-date. If you have any billing related questions, please contact our Author Billing department directly at authorbilling@plos.org.

If your institution or institutions have a press office, please notify them about your upcoming paper to help maximize its impact. If they’ll be preparing press materials, please inform our press team as soon as possible -- no later than 48 hours after receiving the formal acceptance. Your manuscript will remain under strict press embargo until 2 pm Eastern Time on the date of publication. For more information, please contact onepress@plos.org.

Kind regards,

Asem Surindro Singh, Ph.D

Academic Editor

PLOS ONE

Additional Editor Comments (optional):

Reviewers' comments:

# Editor report: Acceptance letter

**DOI:** 10.1371/journal.pone.0295578.r004

**Authors:** Asem Surindro Singh

8 Jan 2024

PONE-D-23-15181R1 

PLOS ONE

Dear Dr. Parks, 

I'm pleased to inform you that your manuscript has been deemed suitable for publication in PLOS ONE. Congratulations! Your manuscript is now being handed over to our production team.

At this stage, our production department will prepare your paper for publication. This includes ensuring the following:

* All references, tables, and figures are properly cited

* All relevant supporting information is included in the manuscript submission,

* There are no issues that prevent the paper from being properly typeset

If revisions are needed, the production department will contact you directly to resolve them. If no revisions are needed, you will receive an email when the publication date has been set. At this time, we do not offer pre-publication proofs to authors during production of the accepted work. Please keep in mind that we are working through a large volume of accepted articles, so please give us a few weeks to review your paper and let you know the next and final steps. 

Lastly, if your institution or institutions have a press office, please let them know about your upcoming paper now to help maximize its impact. If they'll be preparing press materials, please inform our press team within the next 48 hours. Your manuscript will remain under strict press embargo until 2 pm Eastern Time on the date of publication. For more information, please contact onepress@plos.org.

If we can help with anything else, please email us at customercare@plos.org.

Thank you for submitting your work to PLOS ONE and supporting open access. 

Kind regards, 

PLOS ONE Editorial Office Staff

on behalf of

Dr. Asem Surindro Singh 

Academic Editor

PLOS ONE
